# Dr. Mitchill's Description of His Own Case of Croup

**Published:** 1812-03

**Authors:** Sam. L. Mitchill


### *Dr. Mitchill's Description of his own Case of Croup.*

DEAR SIR, *New York, August* 23, 1810.

In answer to the queries proposed in your letter of the 20th instant, concerning the occurrence of croup in adult persons, I offer you a brief account of my own case. I regret that some notice of it, in the newspapers of the day, cannot now be found.

In the spring of the year 1801, I suffered a violent attack of a disease in the trachea, which I have no hesitation to call croup. I am not conscious of any particular exposure or accident that brought it on. Its commencement was more like catarrh than any other disorder. In its progress there was an extraordinary secretion of slime. Very little of this came from the pharynx, fauces, or posterior nares. It proceeded from the glottis, larynx, and trachea. Scarcely any portion of it was secreted below the entrance of the latter into the thorax. The quantity was excessive. It might almost be imagined that all the fluids of my body had rushed, with one onset, to these parts. The toughness of the phlegm rendered it very difficult for me to dislodge it. There was in it a manifest tendency to thicken and concrete. This disposition might have been increased by absorption and evaporation.

Though the mucus of this tenacious quality was so profusely poured out, there was no great irritation in the parts which it inundated. By this I mean, that I was not under any necessity to cough constantly. Had there been no other evacuation than that arising from the *involuntary* action of the diseased organ, I must very soon have been suffocated. The fluids would have accumulated and thickened, and

---

eventually eventually it might have been impossible to have cleared away the adhesive and inspissated mass.

The exercise of my will, alone, saved me from a stoppage of my breathing. Knowing the consequences of a stagnation and concretion of the fluids in the windpipe, I exerted myself, with all my powers, to keep the passage open. My efforts to hawk were exceedingly laborious, and almost incessant. The quantity voided by spitting was enormous. By calling into action all the muscles over which I had any volition, I promoted the discharge. I have no doubt that by practice I acquired the power of making the muscles co-operate more exactly in dislodging the secretion; and I aided the internal voluntary efforts by the application of my fingers to the outside of the assailed parts; by a well-directed pressure, the mucus adhering to the inside of the trachea and larynx was brought more within the current of the expelled air, and was in greater quantity expelled with it. I withheld an epispastic, because I thought it would interrupt the use of the laryngeal muscles, and at the same time prevent the employment of my fingers. I am the more inclined to suppose my disease the croup, inasmuch as I had no soreness nor swelling of my throat, nor was I sensible either of the chills or heats of a fever; my lungs were not oppressed with inflammatory action, and my breathing, as far as the moving powers of the chest and the action of the lungs themselves were concerned, was performed as freely as ever.

The remedies which I employed in so formidable a disease were few. I had the aid of cathartics; I rigidly abstained from all manner of food and drink, until my symptoms abated. I was bled once from the arm by my friend, Dr. V. Seaman. But my principal dependence was placed on the excretion of the overflowing humors, under the direction of my will; and, without this, I do not know how I could have lived many hours. Had an infant, a child, or any person unable to have cleared the larynx, been seized as rudely as I was, death must have ensued, after a series of symptoms which almost every practitioner would have ascribed to cynanche trachealis. This disease has a name more significant and appropriate than usual; for it is really, as its generic and specific terms import, a malady "*which* kills by strangling, or intercepting the passage of the air to and from the lungs through the windpipe." The reason therefore is apparent wherefore grown persons are not more frequently the victims of croup. They relieve themselves by hawking and spitting, and partly also by coughing and raising; and if infants and children could detach the tenacious fluids in the the same way, there would be fewer instances of mortality among them. This is fully confirmed by the recoveries which happen in consequence of sometimes dislodging the secreted substance, even after it has acquired the firmness of a membrane.

It appears to me from the observations I have made on croup, that adults have no exemption from it; they suffer the disease more frequently than is generally supposed; but among this class of patients the fatal termination is more rare, on account of their ability to extricate from the trachea its mordid lining. Notwithstanding which, there occurs now and then a case, wherein, from a want probably of expectorating power, the croup destroys even adults. Hence we understand how the disease, which in an individual of tender age might be croup, is in a person of advanced years generally denominated a cold, and confounded with the diversified forms of catarrh.

This sentiment I had entertained from my own observations and sufferings, before you suggested to me that it had been countenanced by Michaelis. Though I remember that gentleman, I had no recollection of the passage in his dissertation on the membranous or polypose angina, which ascribes the greater *fatality* of the croup in children to their inability to throw off the secreted fluid, and attributes greater *frequency* of it to adults than is generally believed, the latter excreting it before it forms a film or membrane.

This disease, as it occurs in children, and progresses from bad to worse, through all its stages, presents, as you know, a most afflicting spectacle. I shall never forget the incipient hoarseness, the sonorous wheezing, the playful intervals, the distressing croaking, the suffocating anguish, and the premature death, of Robert, the fifth son of my most excellent mother, who, at the age of five years, was overpowered by the accumulation of fluids in the trachea, too viscid for him to bring up.

Wishing you success in your investigation, I remain, with much esteem and regard, your's,

SAM. L. MITCHILL.

*To* Dr. DAVID HOSACK.

COLLEC-

